# Distinguishing reflex from non-reflex responses elicited by transcutaneous spinal stimulation targeting the lumbosacral cord in healthy individuals

**DOI:** 10.1007/s00221-024-06790-2

**Published:** 2024-02-28

**Authors:** Elizabeth A. Gordineer, Dobrivoje S. Stokic, Matthias J. Krenn

**Affiliations:** 1 School of Graduate Studies in the Health Sciences, Program in Neuroscience, University of Mississippi Medical Center, Jackson, MS USA; 2 Center for Neuroscience and Neurological Recovery, Methodist Rehabilitation Center, Jackson, MS USA; 3 Department of Neurosurgery, University of Mississippi Medical Center, 2500 N State St, Jackson, MS 39216 USA

**Keywords:** Electrical stimulation, Evoked motor response, Neurophysiology, Post-activation depression, Spinal reflex, Transcutaneous spinal cord stimulation

## Abstract

Transcutaneous spinal stimulation (TSS) studies rely on the depolarization of afferent fibers to provide input to the spinal cord; however, this has not been routinely ascertained. Thus, we aimed to characterize the types of responses evoked by TSS and establish paired-pulse ratio cutoffs that distinguish posterior root reflexes, evoked by stimulation of afferent nerve fibers, from motor responses, evoked by stimulation of efferent nerve fibers. Twelve neurologically intact participants (six women) underwent unipolar TSS (cathode over T11-12 spinal processes, anode paraumbilically) while resting supine. In six participants, unipolar TSS was repeated 2–3 months later and also compared to a bipolar TSS configuration (cathode 2.5 cm below T11-12, anode 5 cm above cathode). EMG signals were recorded from 16 leg muscles. A paired-pulse paradigm was applied at interstimulus intervals (ISIs) of 25, 50, 100, 200, and 400 ms. Responses were categorized by three assessors into reflexes, motor responses, or their combination (mixed responses) based on the visual presence/absence of paired-pulse suppression across ISIs. The paired-pulse ratio that best discriminated between response types was derived for each ISI. These cutoffs were validated by repeating unipolar TSS 2–3 months later and with bipolar TSS. Unipolar TSS evoked only reflexes (90%) and mixed responses (10%), which were mainly recorded in the quadriceps muscles (25–42%). Paired-pulse ratios of 0.51 (25-ms ISI) and 0.47 (50-ms ISI) best distinguished reflexes from mixed responses (100% sensitivity, > 99.2% specificity). These cutoffs performed well in the repeated unipolar TSS session (100% sensitivity, > 89% specificity). Bipolar TSS exclusively elicited reflexes which were all correctly classified. These results can be utilized in future studies to ensure that the input to the spinal cord originates from the depolarization of large afferents. This knowledge can be applied to improve the design of future neurophysiological studies and increase the fidelity of neuromodulation interventions.

## Introduction

Activation of sensory fibers is a common approach to neuromodulation. Transcutaneous spinal stimulation (TSS) has been introduced as a technique for depolarizing posterior roots over the lumbosacral or cervical segments and providing input to the spinal cord. In neurophysiological studies, TSS is often paired with another spinal or supraspinal input to deduce the magnitude and time course of neuromodulation at the spinal or cortical level (Knikou 2014; Pulverenti et al. 2021; Roy et al. 2014; Shapkova 2004; Shapkova and Schomburg 2001). As a therapeutic modality, TSS can produce short-term changes in spinal excitability that are believed to promote the reorganization of neural pathways (de Freitas et al. 2021). Based on this assumption, TSS has been used in attempts to enable movements (Inanici et al. 2021), reduce spasticity (Hofstoetter et al. 2021), and improve autonomic functions (Samejima et al. 2022) after spinal cord injury in humans, whether used alone (Megía-García et al. 2021; Meyer et al. 2020) or in combination with other rehabilitation strategies (Al’joboori et al. 2020; Gad et al. 2017; Hofstoetter et al. 2021; Inanici et al. 2021).

For TSS to exert neuromodulation effects, the predominant if not exclusive, depolarization of afferent fibers is essential. Yet, lumbosacral and cervical TSS can evoke not only reflex responses but also motor responses due to motor fiber depolarization, as well as mixed responses showing features of both reflex and motor responses (Binder et al. 2021; Danner et al. 2016; Roy et al. 2012; Wu et al. 2020). Reflex and non-reflex responses have been distinguished based on the difference in onset latencies (Danner et al. 2016; Minassian et al. 2007) or with conditioning paradigms (Roy et al. 2012; Saito et al. 2019). Among the latter, a paired-pulse paradigm at different interstimulus intervals (ISIs) is most commonly used. If the second response in the pair is suppressed at short ISIs, the evoked responses are believed to be of reflex origin (Andrews et al. 2015; Courtine et al. 2007; Minassian et al. 2007; Roy et al. 2012).

Consistent with experimental findings, computer simulation studies support the possibility of evoking reflex and motor responses with both epidural stimulation and TSS. An epidural model has proposed that with a posterior midline electrode location, the threshold for depolarizing posterior roots is lower than for depolarizing anterior roots (Rattay et al. 2000). TSS models have suggested that for posterior roots, low threshold sites are located just before they enter into the spinal cord and as they exit the spinal canal where anterior roots can also be activated (Ladenbauer et al. 2010). Another model has suggested that with increasing TSS intensities, the order of activation is posterior roots, anterior roots, and posterior columns (Danner et al. 2011). The latter authors also proposed that identifying TSS responses is essential to properly interpret neurophysiologic studies examining the conducting and processing capabilities of the spinal cord and understanding the neurophysiology behind the putative effects of neuromodulation interventions.

Despite recognizing that TSS may evoke both reflex and motor responses, previous reports did not investigate in detail their prevalence, coexistence, repeatability, associated paired-pulse ratios, or the impact of different electrode montages. Therefore, the main objectives of this study were to identify different response types elicited by unipolar TSS, determine their prevalence, and establish paired-pulse ratio cutoffs at different ISIs that can reliably distinguish reflex from non-reflex responses. We then evaluated the validity of the derived paired-pulse ratio cutoffs when repeated 2–3 months later and when applied to a bipolar electrode configuration.

## Materials and methods

### Participants

Twelve participants (six women) with no history of neurological disorders were recruited for this study. Their average (standard deviation) age was 30.3 (9.8) years (range 24–59), height was 174.6 (8.3) cm (range 158–188), and body mass was 75.8 (14.1) kg (range 59–95). The study was approved by the Institutional Review Board Committee of the University of Mississippi Medical Center, Jackson, MS (UMMC-IRB 2020-0193). All participants signed written informed consent before their enrollment into the study.

### Experimental procedure

The study consisted of two sessions separated by 74 (6) days. All 12 participants were included in the first session (S1), which aimed to identify different response types elicited by unipolar TSS, determine their prevalence, and establish the optimal cutoff point(s) for their discrimination. In the second session (S2), six participants (two women) from the initial cohort were available to validate the developed approach for distinguishing reflex from non-reflex responses using the same unipolar TSS configuration and additionally a bipolar configuration.

During data collection, the participants were lying supine on a hospital bed. The supine position was preferred over the prone position as it predominantly recruits afferent fibers (Danner et al. 2016). We ensured a controlled and comfortable environment that minimized distractions. Participants were instructed not to talk or move during recordings.

### TSS procedure

#### Electrode configurations

For the unipolar configuration (heretofore sessions UP_S1_ and UP_S2_), a self-adhesive surface electrode (5 × 5 cm) serving as the cathode was placed midline over the T11-T12 spinal processes, identified by palpating anatomical landmarks. Four interconnected electrodes (5 × 10 cm each) were placed paraumbilically as a large indifferent reference electrode (anode). In the second session (UP_S2_), the unipolar electrode placement was replicated based on the individual photos taken at UP_S1_. For the bipolar configuration (BP_S2_), two 5 × 5-cm self-adhesive surface electrodes were used, with the cathode placed 2.5 cm below the location used for the unipolar stimulation and the anode placed 5 cm above the cathode (Krenn et al. 2020).

#### Stimulation procedure

Monophasic 1-ms rectangular pulses were delivered by a current-controlled stimulator (DS8R, Digitimer Ltd, Welwyn Garden City, UK). An analog output and acquisition card controlled the stimulation pulse timing and intensity (USB-NI 6003, National Instruments Inc., Austin, TX, USA). The trigger signal for the stimulator was also used as a sync input for the electromyographic (EMG) system. We first selected the stimulation range for each participant using a custom-made real-time viewing program (LabVIEW 2019, National Instruments Inc.).

The stimulation intensities were set based on the range established for each individual. The low end of the range was defined as the intensity that produced a just visible EMG response in any muscle. The high end of the range was defined as the intensity where a 20% increment in intensity produced no further increase in the peak-to-peak amplitude in the soleus muscle. No participant reported discomfort at the selected stimulation intensities. Within this range, we collected 42 data points at incremental intensities to be able to derive the recruitment curve for each muscle.

For the paired-pulse stimulation paradigm, three stimulation intensities were applied corresponding to 25%, 50%, and 75% of the individual stimulation range (for example, in ID03, the established range was 25 to 100 mA; thus, the stimulation intensities applied were 44, 63, and 81 mA). Pulses were delivered at ISIs of 25, 50, 100, 200, and 400 ms with a 5-s pause between consecutive pairs. The order of ISIs was randomized. For each ISI, the stimulation intensity and four repetitions of each intensity were randomized. The protocol deviated for one participant (ID08), in whom ISIs ranged from 50 to 400 ms and were repeated twice.

### EMG recordings

The skin was abraded with a gel before bipolar surface electrodes (2.5 cm inter-electrode distance) were centered over the muscle belly. Twelve electrodes were placed on the leg corresponding to the hand-dominant side: vastus medialis (VM1), vastus lateralis (VL1), rectus femoris (RF1), adductors (AD1), lateral hamstrings (LH1), medial hamstrings (MH1), peroneus longus (PL1), tibialis anterior (TA1), lateral gastrocnemius (LG1), medial gastrocnemius (MG1), soleus (SO1), and extensor digitorum brevis (EB1), and on four muscles of the contralateral side (RF2, MH2, TA2, SO2). The decision to account for hand dominance was based on controversy of previous observations of different excitability in the soleus H-reflex (Nativ et al. 1989; Tan 1985). EMG signals were acquired with a wireless data acquisition system (Noraxon U.S.A. Inc., Scottsdale, AZ, USA). The hardware low-pass filter was set to 2 kHz, and signals were digitized with 16-bit resolution at 4,000 samples per second.

### Data analysis and statistical analysis

#### Calculation of the paired-pulse ratio

##### Response amplitude

The EMG signal was digitally filtered with a second-order, 5-Hz high-pass filter to remove the offset. The peak-to-peak amplitudes of the first and the second response were calculated in the windows individually selected for each participant and muscle. These windows were kept constant over all stimulation intensities, sessions, and electrode configurations. On average, the windows ranged from 9.6 (1.5) to 29.4 (2.8) ms in the proximal muscles and from 15.4 (3.3) to 31.0 (3.2) ms in the distal muscles.

##### Selection of responses for analysis

Out of the three stimulation intensities applied, we included in the UP_S1_ analysis the responses evoked by the stimulation intensity (25, 50, or 75% of the established range) that approximated the steepest part of the recruitment curve for each muscle to make results comparable across muscles. The corresponding stimulation intensity was 82.0 (23.1) mA or 1.42 (0.27) times the motor threshold across all muscles. From the second session, we selected for analysis a stimulation intensity that produces the most comparable amplitudes of the first response between UP_S1_, UP_S2_, and BP_S2_ (average of 80.0 (15.2) mA, 83.0 (15.6) mA, and 105.2 (23.6) mA, respectively). The agreement between amplitudes of the first responses was tested by a two-way random effect, absolute agreement, single measurement intraclass correlation (ICC) model (McGraw and Wong 1996) using the Pingouin 0.5.3 Python package (Vallat 2018). The ICC between response amplitudes of UP_S1_ (2.41 (2.51) mV) and UP_S2_ (2.62 (2.60) mV) was 0.76, with a 95% confidence interval (CI_95%_) from 0.66 to 0.84. Similarly, the average response amplitudes for UP_S2_ and BP_S2_ were 2.60 (2.60) mV and 2.06 (2.40) mV, with an ICC of 0.77, CI_95%_ [0.65, 0.85].

##### Outcome measure

The paired-pulse ratio was calculated for all ISIs as the ratio of the amplitude of the second response to the averaged amplitude of the first responses across 50–400 ms ISIs (25-ms ISI not included due to possible contamination by the second stimulation artifact). Before calculating the average amplitude of the first response, outliers were removed if the value exceeded five times the lower or upper semi-interquartile range measured from the median (Dovoedo and Chakraborti 2015; Schwertman et al. 2004), which occurred in 2.9% (165 of 5,594) of all data points.

##### Missing data

Due to poor signal quality, all recordings for EB1 were discarded. In addition, due to EMG sensor failure in three instances of UP_S1_, no data were recorded in the LH1 (ID12) and PL1 (ID02), and partial data were recorded in the VM1 (ID11), resulting in a loss of 52 data points. Another 13 data points were lost due to miscellaneous errors in the EMG recording. In total, 6,955 out of 7,020 data points (99.1%) were available for analysis.

#### Visual classification of response types

All short-latency responses included in the analysis were visually examined by each author independently. The paired-pulse responses in each muscle were displayed for all ISIs and classified based on their appearance and the degree of suppression into reflex, motor, or mixed responses. Reflexes were defined by an apparent suppression of the second response at short ISIs followed by recovery at increasing ISIs. Motor responses were those where the first and second responses were similar in appearance across ISIs (shape, size). The coexistence of the motor and reflex responses, where the compound waveform is changing in size and/or shape across ISIs, was defined as a mixed response. One response type was assigned to each muscle per individual.

The agreement between the assessors in classifying the response types was tested by Fleiss’s kappa analysis in R version 4.2.3 (R Core Team 2023). The agreement was determined based on the alpha values (range -1 to 1) and interpreted as poor (< 0), slight (0 to 0.2), fair (0.2 to 0.4), moderate (0.4 to 0.6), substantial (0.6 to 0.8) or almost perfect (0.8 to 1) (Nichols et al. 2010). The final dataset of the visual classification included responses that at least two assessors categorized in the same way.

The prevalence (counts and percentages) of response types (reflex, motor, mixed) was calculated for each condition from the final visual classification dataset. We then tested whether the proportions of the identified response types differed between legs (UP_S1_ dominant vs. non-dominant), sessions (UP_S1_ vs. UP_S2_), and montages (UP_S2_ vs. BP_S2_), using 2 × 2 McNemar’s tests in R version 4.2.3 (Pembury Smith and Ruxton 2020; R Core Team 2023).

#### Distinguishing reflex from non-reflex responses based on paired-pulse ratios

The paired-pulse ratios from UP_S1_ were used to establish a numeric criterion for distinguishing between visually classified reflex and non-reflex responses. For this, non-parametric receiver-operating characteristic (ROC) curves were created at each ISI for all muscles combined. The curves were generated using the “ROC Analysis” procedure in SPSS statistical software (version 29, IBM Corp., Armonk, NY, USA). The software provides a true positive rate (sensitivity) and false positive rate (1-specificity) for each ROC model. The discriminative performance of different models at each ISI was compared by the area under the curve (AUC) and CI_95%_.

The optimal cutoff value of each ROC model was determined as the paired-pulse ratio corresponding to the maximized Youden’s J statistic (Youden 1950). In addition, the macro-average accuracy of response type classifications was calculated based on the decision matrices, which report the true positives and negatives as well as the falsely classified responses in either direction.

##### Validation of derived cutoff values

The cutoffs from UP_S1_ were applied to the UP_S2_ data to assess their validity over time (sessions). The same procedure was repeated with the BP_S2_ data to determine their validity for the bipolar electrode configuration. The decision matrices and accuracy of classification for UP_S2_ and BP_S2_ were reported.

## Results

### Types and prevalence of responses elicited by unipolar lumbosacral TSS

**Fig. 1:**
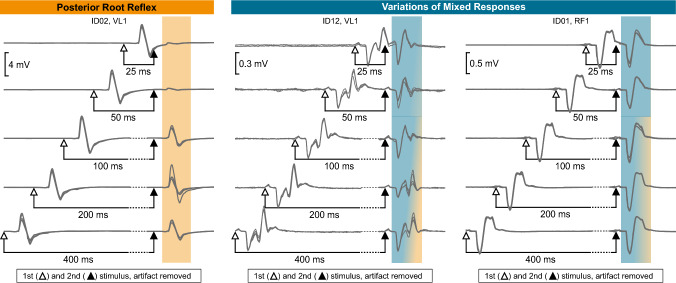
Short-latency response types. Examples of posterior root reflex responses (left) and variations of mixed responses ranging from about equal motor and reflex components (center) to predominant motor component (right) in different participants (ID02, ID12, ID01) and muscles (VL1, RF1). Four responses are superimposed at each interstimulus interval (25–400 ms). The open and closed arrows indicate the timing of delivery of the first and second stimulation pulse (stimulation artifact removed for better visualization). Shaded areas highlight the second response, and the presence of reflex and motor components is shown in grades of orange and blue, respectively. *VL1* vastus lateralis, *RF1* rectus femoris

Out of three defined response types, all three assessors identified only reflex and mixed responses but no sole motor responses. Reflexes showed a clear suppression of the second response at short ISIs with recovery at longer ISIs (Fig. 1, left). Mixed responses showed a more complex waveform. Although the motor (first) and reflex (second) components were largely overlapping, they were visually distinguishable. The size of the reflex component ranged from being comparable to the motor component (Fig. 1, middle) to being relatively small (Fig. 1, right). Still, the reflex component showed apparent suppression at shorter ISIs, whereas the motor component did not.

In independent visual analysis, the three assessors identically classified 377 out of 384 (98.2%) responses over all muscles, participants, sessions, and montages. Pairwise agreements between assessors ranged from 98.3 to 99.4%. The Fleiss’s kappa for the three assessors was 0.90, suggesting almost perfect agreement.

**Fig. 2:**
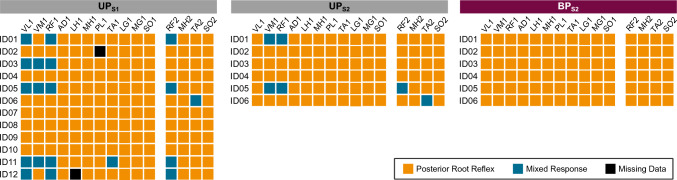
Final visual classification of response types by the three assessors. Posterior root reflexes (orange) and mixed responses (blue) are denoted across different muscles for each participant (ID01–ID12). Of the 12 participants included in the first session (S1), 6 also participated in the second session (S2). TSS was applied in unipolar (UP) and bipolar (BP) electrode configurations. *VM* vastus medialis, *VL* vastus lateralis, *RF* rectus femoris, *AD* adductors, *LH* lateral hamstrings, *MH* medial hamstrings, *PL* peroneus longus, *TA* tibialis anterior, *LG* lateral gastrocnemius, *MG* medial gastrocnemius, *SO* soleus [1, dominant side; 2, non-dominant side]

The result of the final visual classification of responses by all assessors together is presented in Fig. 2. In UP_S1_, the prevalence of reflexes across 15 muscles and 12 participants was 90% (160 out of 178, responses in 2 muscles were missing due to sensor error). Consequently, the overall prevalence of mixed responses was 10% (18/178). They were observed in only four muscles, of which in two bilaterally (prevalence per muscle: VL1, 33%; VM1, 25%; RF1, 42%; RF2, 33%; TA1, 8%, and TA2, 8%). When analyzing the concordance of response types between bilaterally recorded muscles (RF, MH, TA, SO), the same response type was observed in 45 of 48 instances (94%), whereas only in 3 (6%) instances, response type changed from reflex to mixed responses. McNemar’s test showed that the conversion from reflex to mixed responses was not significant between the bilaterally recorded muscles (*X*^2^(1) = 0.33, *p* = 0.56).

### Profiles of paired-pulse suppression of reflexes and mixed responses

**Fig. 3:**
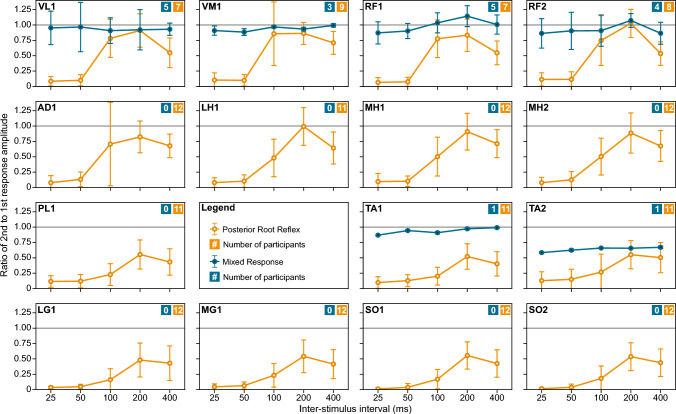
Paired-pulse profiles of posterior root reflexes and mixed responses in 12 participants. The group means (circles) and standard deviations (error bars) of the paired-pulse ratios for reflexes (orange) and mixed responses (blue) recorded in UP_S1_ are shown for different muscles and interstimulus intervals. The number of participants contributing to each type of response is indicated in the top right corner of each panel. Muscle abbreviations as in Fig. 2

Figure 3 shows the mean paired-pulse ratios elicited by UP_S1_ for visually classified reflexes and mixed responses over 25–400-ms ISIs in all recorded muscles. For reflexes, the paired-pulse suppression was profound at shorter ISIs in all muscles, with faster recovery in the proximal than distal muscles. As expected, the greatest suppression of reflexes was observed at short ISIs with median paired-pulse ratios of 0.041 (interquartile range, IQR: 0.002, 0.093) at 25-ms ISI and 0.060 (IQR: 0.023, 0.132) at 50-ms ISI. Mixed responses showed little to no variation in paired-pulse suppression across ISIs with median paired-pulse ratios of 0.889 (IQR: 0.678, 1.028) at 25-ms ISI and 0.889 (IQR: 0.691, 1.035) at 50-ms ISI. In general, the paired-pulse ratios at 100- and 200-ms ISIs were larger than those at 400-ms ISI.

### Paired-pulse ratio cutoffs for distinguishing reflexes from mixed responses

**Fig. 4:**
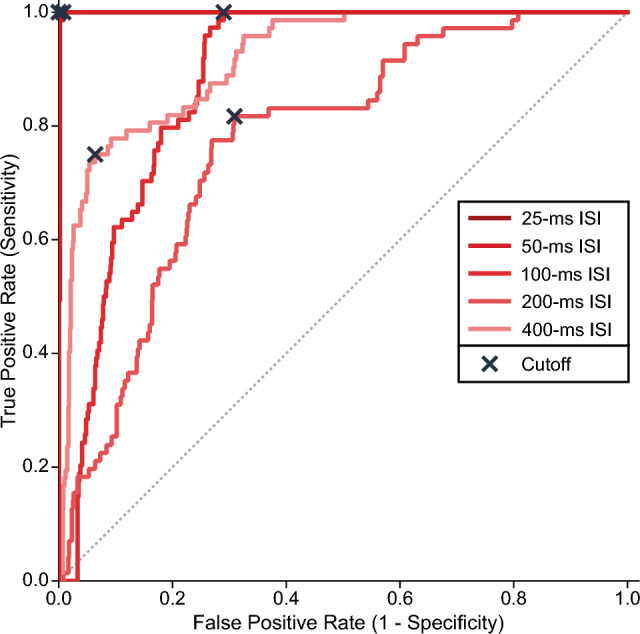
The receiver-operating characteristic curves of paired-pulse ratios recorded in UP_S1_ for each interstimulus interval (ISI). The cutoff point for each curve is indicated by an X. Corresponding ROC metrics are presented in Table 1. The data for all participants and muscles were used to generate the ROCs

The ROC analysis provided the best cutoff point that separated reflexes from mixed responses at each ISI (Fig. 4). The best results were obtained at ISIs of 25 ms (AUC = 1.000) and 50 ms (AUC = 0.999), whose confidence intervals overlapped (Table 1). In contrast, the AUC for ISIs of 100, 200, and 400 ms was lower (0.885, 0.772, 0.918), and their confidence intervals were outside of the CI_95%_ of both 25- or 50-ms ISI.

**Table 1:** Metrics of the receiver-operating characteristic curve for each interstimulus interval for UP_S1_

ISI (ms)	AUROC (CI_95%_)	Optimal cutoff values			
		Paired-pulse ratio	Accuracy (%)	Sensitivity (%)	Specificity (%)
25	1.000 (1.000, 1.000)	0.512	100	100.0	100.0
50	0.999 (0.997, 1.001)	0.470	97	100.0	99.2
100	0.885 (0.859, 0.912)	0.594	65	100.0	71.0
200	0.772 (0.721, 0.824)	0.899	60	81.7	69.1
400	0.918 (0.888, 0.948)	0.869	77	75.0	93.6

*AUROC* area under the receiver-operating characteristic curve, *CI*_*95%*_ 95% confidence interval, *ISI* interstimulus interval. Accuracy refers to macro-average accuracy

**Fig. 5:**
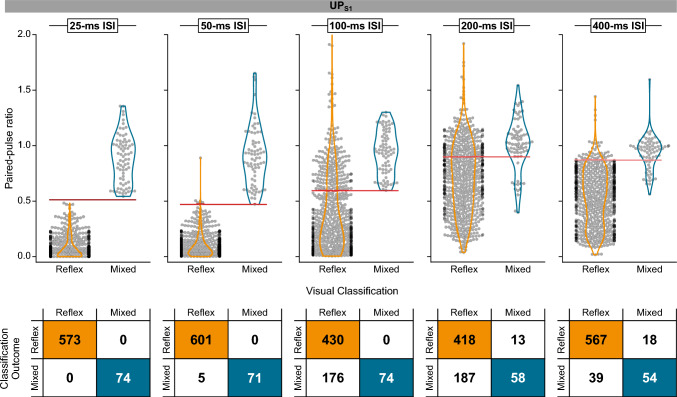
Paired-pulse ratios for reflexes and mixed responses relative to the cutoff values derived from UP_S1_. Scatterplot of data points (top) and decision matrices (bottom) for different interstimulus intervals (ISIs) for all muscles. Horizontal lines (red) indicate the cutoff value for each ISI

The optimal cutoff value of paired-pulse ratios at 25-ms ISI was 0.512. This cutoff correctly classified all 647 reflex and mixed responses (100% sensitivity and specificity) (Table 1). Similarly, at 50-ms ISI, the cutoff value was 0.470, with 672 correctly classified reflex and mixed responses and only 5 reflexes misclassified as mixed responses (100% sensitivity, 99% specificity). As expected, the sensitivity and specificity values were lower at ≥ 100-ms ISI due to less reflex suppression, resulting in greater overlap with mixed responses. Figure 5 shows the distribution of reflex and mixed responses below and above the derived cutoff values at different ISIs and the corresponding decision matrices.

### Validation of cutoff values between sessions and electrode configurations

In a repeated session with six participants (UP_S2_), the prevalence of visually classified reflexes and mixed responses was 93% (84/90) and 7% (6/90) (Fig. 2). Mixed responses were observed in four muscles (VM1, 33%; RF1, 33%; RF2, 17%; and TA2 17%). The concordance of reflexes and mixed responses between the sessions UP_S1_ and UP_S2_ was 92% (82/89), with only 8% (7/89) of responses changing from reflex to mixed type and vice versa. This rate of change was not significant (McNemar’s test: *X*^2^(1) = 3.57, *p* = 0.06).

**Fig. 6:**
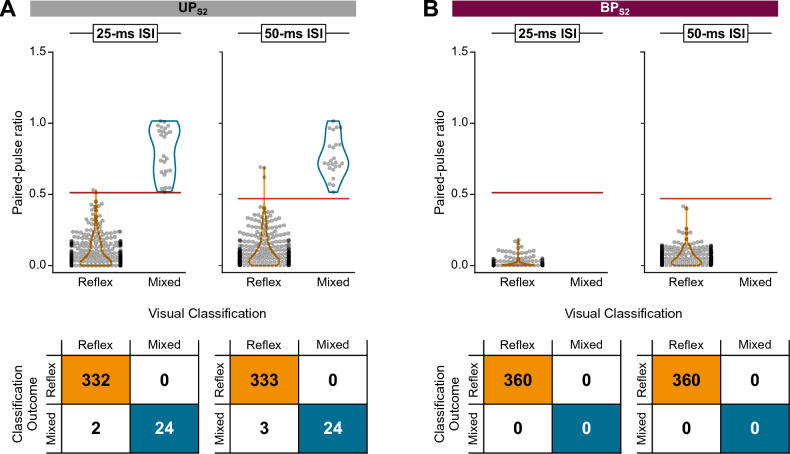
Paired-pulse ratios for reflexes and mixed responses from UP_S2_ and BP_S2_ relative to the cutoff values derived from UP_S1_. Scatterplot of data points (top) and decision matrices (bottom) for different interstimulus intervals (ISIs) for UP_S2_ (**A**) and BP_S2_ (**B**) for all recorded muscles. Cutoff values from UP_S1_ are indicated by the horizontal lines (red) for each ISI

Next, we used the derived cutoff values of 0.512 (25-ms ISI) and 0.470 (50-ms ISI) from UP_S1_ to determine their validity when applied to UP_S2_ (Fig. 6A). For the 0.512 (25-ms ISI) cutoff, 356 reflex and mixed responses were correctly classified with only 2 reflex responses misclassified (99% accuracy). For the 0.470 (50-ms ISI) cutoff, 357 reflex and mixed responses were correctly classified and only 3 reflex responses were misclassified (99% accuracy). Both cutoff values performed well and comparably in terms of sensitivity (100% for both) and specificity (> 89%).

**Fig. 7:**
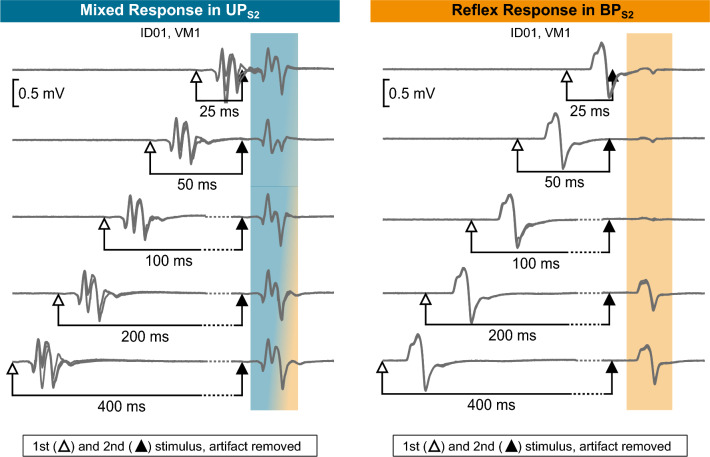
Example of a recorded mixed response in UP_S2_ and reflex response in BP_S2_ for participant ID01 and VM1 muscle. Four responses are superimposed at each interstimulus interval (25–400 ms). The open and closed arrows indicate the timing of delivery of the first and second stimulation pulse (stimulation artifact removed). Shaded areas highlight the second response, and the presence of reflex and motor components is shown in grades of orange and blue, respectively. *VM1* vastus medialis

BP_S2_ exclusively elicited reflex responses (Fig. 2). Thus, six instances (7%) of mixed responses elicited by UP_S2_ converted to reflexes with BP_S2_ (example shown in Fig. 7). This conversion from mixed responses in UP_S2_ to reflex responses in BP_S2_ was significantly different (McNemar’s test: *X*^2^(1) = 6, *p* = 0.014). As to the discriminative ability of the derived cutoff values, both 0.512 (25-ms ISI) and 0.470 (50-ms ISI) cutoffs correctly identified all responses as reflexes with 100% sensitivity and specificity (Fig. 6B).

## Discussion

The results of this study indicate that unipolar lumbosacral TSS mainly but not exclusively evokes reflex responses. In our sample, motor responses were never elicited in isolation but always in conjunction with reflexes, which were suppressed to various degrees. These mixed responses were identified mainly in the quadriceps muscles. Using the visual classification as a reference, we determined that ISIs of 25 and 50 ms are best suited for distinguishing reflexes from mixed responses. The corresponding paired-pulse ratios of 0.51 (25-ms ISI) and 0.47 (50-ms ISI) can reliably discriminate reflex from non-reflex responses. The established cutoffs performed well between repeated sessions separated by 2–3 months. Bipolar TSS exclusively elicited reflexes that were all correctly classified by the derived cutoff values.

### Response types elicited by TSS

Early studies demonstrated the possibility of evoking motor responses besides reflexes with a cathode at different locations over the spinal column (T11 to S1) and the anode over the iliac crest contralateral to the leg being examined with high voltage stimulation pulses (Maertens de Noordhout et al. 1988; Troni et al. 1996). More recently, Minassian et al. (2007) reported the presence of motor responses in the quadriceps muscle (seemingly rectus femoris) in one out of eight participants evaluated in the supine position with unipolar TSS (cathode over T11-T12 interspinous space). Our results somewhat differ from Minassian et al. (2007) because we never identified motor responses in isolation but always overlapped with reflexes. In a few instances, however, the visible suppression of the reflex component was rather small, which could be misinterpreted as a lone motor response.

Previous TSS studies have applied different procedures in an attempt to elicit only reflexes, such as adjusting stimulation intensities (Sayenko et al. 2015), moving the stimulation electrodes (Skiadopoulos et al. 2022), and placing the participant in the supine position (Saito et al. 2019). Sayenko et al. (2015) used lower stimulation intensities to minimize the spread of current to motor fibers; however, if the goal is to elicit prominent responses in the quadriceps muscle, higher stimulation intensities are necessary, possibly resulting in mixed responses, as shown here. Searching for the optimal electrode position during recordings is not necessarily difficult but can be time-consuming. Roy et al. (2012) showed that stimuli over L3 elicited markedly suppressed second responses, whereas the same stimulation over S1 produced identical first and second responses, indicating activation of motor axons. Other studies have adopted this approach of adjusting the electrode placement to ensure clear suppression of the second response, suggesting their reflex origin (Skiadopoulos et al. 2022). As to placing the subject in the supine position, Danner et al. (2016) demonstrated that while the prone position predominantly evoked motor or mixed responses, reflex responses were mainly elicited in the supine. In standing, Binder et al. (2021) reported motor responses in the rectus femoris if the spine was flexed, whereas mixed responses were evoked if the spine was extended or in a neutral position. Collectively, optimizing the study protocol could reduce the occurrence of mixed responses, but eliminating them entirely in some muscles is unlikely with unipolar TSS. Thus, mixed responses may need to be accounted for and ideally excluded from analysis if the intent is to draw conclusions based on modification of reflexes.

### Paired-pulse paradigm as a discriminator of response types

Paired-pulse paradigm has been used to assess the nature of responses evoked by TSS and account for the possibility of eliciting motor (Danner et al. 2016; Minassian et al. 2007) and mixed responses (Binder et al. 2021; Danner et al. 2016; Saito et al. 2019) in addition to reflexes. However, most TSS reports have an incomplete description of the approach used for ensuring the collected or analyzed data are limited to reflexes. Typically, this description refers to using paired pulses at ISIs from 30–100 ms, but it lacks specific details or criteria other than observing the suppression of the second response before commencing an experiment. However, relying solely on visual suppression may be insufficient because, as shown here, mixed responses can also be suppressed to a variable degree owing to the suppression of the reflex component. Indeed, when we analyzed mixed responses and reflexes in aggregate, the paired-pulse ratios (e.g., UP_S1_ 50-ms ISI in VL1: 0.47, RF1: 0.43, MH1: 0.10, TA1: 0.20, SO1: 0.04) agree with those reported in many previous studies (Courtine et al. 2007; Danner et al. 2016; Hofstoetter et al. 2019; Sayenko et al. 2015). Lesser suppression of mixed responses, commonly observed in the proximal muscles as reported here and previously (Roy et al. 2012; Saito et al. 2021), may accidentally confound the results and lead to different interpretations compared to profound suppression of reflexes exclusively evoked in other muscles.

A few studies have outlined specific criteria for defining reflex responses, such as “complete attenuation” of the second response at 50-ms ISI in the SO and TA muscles (Andrews et al. 2015) or inclusion of responses if the second one was below 100 µV peak-to-peak at 50-ms ISI in any of the recorded muscles (Saito et al. 2019). The complete attenuation criterion, presumably judged online, may not generally be applicable because even reflexes in our study showed, in aggregate, an incomplete suppression at 50-ms ISI across all studied muscles (median paired-pulse ratio 0.060, IQR: 0.023, 0.132). At the same time, our results suggest that even less than complete attenuation would still be sufficient to differentiate reflexes from mixed responses, thereby validating this rather vague criterion. As to the 100-µV criterion adopted by Saito et al. (2019) based on the results of Minassian et al. (2007), we reanalyzed our data at 50-ms ISI and found that this criterion is rather stringent. This is because 35% (212/606) of the visually identified reflexes would have to be excluded, potentially reducing the available data in some muscles. On the other hand, the same criterion misclassified only 10% of mixed responses as reflexes. Collectively, these two criteria would perform well in discriminating reflexes from mixed responses but at the cost of excluding a sizable proportion of reflexes.

Currently, there is no consensus on which ISI best discriminates reflex from non-reflex responses. Most studies use 50-ms ISI (Hofstoetter et al. 2008; Minassian et al. 2007; Roy et al. 2012; Sayenko et al. 2015) to avoid overlap of responses and stimulus artifacts and, at the same time, ensure the most prominent post-activation depression. Our ROC results support that choice since paired-pulse ratios at 25- and 50-ms ISI were the most capable of differentiating reflexes from mixed responses. To avoid contamination of the first response by the second stimulation artifact at 25 ms, we recommend using an ISI in the 30–50 ms range for this purpose.

The most salient finding of our study is the possibility of distinguishing reflexes from mixed responses based on paired-pulse ratios at 25- and 50-ms ISI. Paired-pulse ratios of 0.512 (25-ms ISI) and 0.470 (50-ms ISI) best discriminated reflexes from mixed responses with high sensitivity (100.0%) and specificity (> 99.2%). As expected, the differentiation between the two response types was less successful at ISIs of 100–400 ms, as evident by the decrease in sensitivity and specificity, which is due to the overall lesser paired-pulse suppression of reflexes at longer ISIs (Fig. 3). The cutoff values at 25- and 50-ms ISI were validated when applied to the second session 2–3 months later in the subgroup of initial participants as demonstrated by the negligible rate of misclassified reflexes and mixed responses (Fig. 6). This suggests that derived paired-pulse cutoffs can be utilized over repeated sessions of unipolar TSS in the configuration used here. Furthermore, the same cutoffs were also validated on reflex responses exclusively evoked by bipolar TSS. The bipolar paired-pulse ratios were well below the derived cutoffs resulting in no misclassification of reflexes as mixed responses (Fig. 6). Thus, responses with paired-pulse ratios less than 0.47 (lower limit of CI_95%_ for both 25- and 50-ms ISI) can be considered reflexes with high confidence, whether collected over time or evoked by unipolar or bipolar TSS, as used here.

### Limitations

This study has some potential limitations. The results were based on the unipolar TSS configuration with a cathode placed over T11-12 spinal processes. However, this configuration is commonly used, so the results are expected to be applicable to many TSS studies. Although the second session had a smaller sample size, the participants were recruited based on their availability and without a priori knowledge of the results of the first session. Even in this smaller sample, mixed responses were found mainly in the same subjects and muscles, providing confidence in the results. Moreover, the cutoff values derived from the larger sample were successfully validated in the smaller sample by repeating the recording with the same unipolar configuration and extended to the bipolar configuration. The cutoff values were derived by combining responses recorded in different muscles rather than for each muscle separately. Nonetheless, the distribution of the paired-pulse ratios of both reflex and mixed responses at 25- and 50-ms ISI was similar across muscles (Fig. 3), suggesting that individual cutoffs would not differ much from those obtained after pooling the responses. The response amplitudes were analyzed as peak-to-peak values, yet this is advantageous compared to the area under the curve when it comes to the overlapping responses in which different phases can cancel each other. Finally, the cutoff values are derived from neurological intact individuals, which may be considered too restrictive when applied to an injured population, such as after a spinal cord injury, because of their comparably smaller suppression and faster recovery (de Freitas et al. 2021; Hofstoetter et al. 2019). However, the reported paired-pulse ratios are below 0.1 (Knikou and Murray 2019) and 0.2 (Hofstoetter et al. 2019) at ISI at 60 ms; therefore, the established criterion of 0.5 would still likely identify all reflex responses.

## Conclusion and implications

We provide evidence that the commonly used unipolar lumbosacral TSS can elicit not only reflexes but also mixed responses consisting of motor and reflex components, mainly in the quadriceps muscles. Reflexes can be distinguished from mixed responses by paired-pulse ratios rounded to 0.5 at 25- and 50-ms ISI. The paired-pulse cutoff is valid when applied over time and for both unipolar and bipolar TSS configurations.

Our findings translate into a simple quantitative approach for ensuring that the input to the spinal cord provided by TSS originates from the depolarization of large afferents. This is important for neurophysiological studies that use TSS to deliver conditioning or test stimuli as well as for neuromodulation interventions whose goal is to modify excitability and engage circuits at the spinal and supraspinal levels. The depolarization of motor fibers would be counterproductive in both situations, possibly causing the collision of descending and ascending volleys within the motor fibers, antidromic depolarization of motor neurons (like *F*-wave), and the possibility of producing recurrent inhibition. This could confound the interpretation of results in neurophysiological studies and, regarding neuromodulation, alter the state of excitability of the spinal cord in a non-intended manner.

To apply the proposed approach for identifying reflexes, paired-pulse suppression should be quantified. This can be done in preparation for TSS experiments and interventions to ensure adequate TSS input or by eliminating non-reflex responses during data processing before proceeding with the analysis. As a result, future studies would gain greater confidence in conclusions and increase the fidelity of neuromodulation interventions.

## Data Availability

The data that support the findings of this study are available from the corresponding author upon reasonable request.
